# Prognostic impact of Dynamin related protein 1 (Drp1) in epithelial ovarian cancer

**DOI:** 10.1186/s12885-020-06965-4

**Published:** 2020-05-24

**Authors:** Hideaki Tsuyoshi, Makoto Orisaka, Yuko Fujita, Meshach Asare-Werehene, Benjamin K. Tsang, Yoshio Yoshida

**Affiliations:** 1 Department of Obstetrics and Gynecology, University of Fukui, 23-3 Matsuoka-Shimoaizuki, Eiheiji-cho, Yoshida-gun, Fukui, 910-1193 Japan; 2 Departments of Obstetrics & Gynecology and Cellular & Molecular Medicine, and Interdisciplinary School of Health Sciences, University of Ottawa, Ottawa, ON K1H 8L1 Canada; 3 Chronic Disease Program, Ottawa Hospital Research Institute, Ottawa, ON K1H 8L6 Canada

**Keywords:** Epithelial ovarian cancer, Drp1, Phospho-Drp1^Ser637^, CaMKI, Prognostic biomarker

## Abstract

**Background:**

The mitochondrial fission protein, Dynamin related protein 1 (Drp1), and its upstream protein calcium/calmodulin–dependent protein kinase I (CaMKI) play a critical role in chemoresistance in ovarian cancer (OVCA). Thus, we examined the expression of Drp1, CaMKI and their phosphorylated forms and their prognostic impact in epithelial OVCA patients.

**Methods:**

Expression analysis was performed by immunohistochemistry (IHC) of paraffin-embedded tumor samples from 49 patients with epithelial OVCA. Staining intensity and the percentage of positively stained tumor cells were used to calculate an immunoreactive score (IRS) of 0–12. The expression scores calculated were correlated with clinicopathological parameters and patient survival.

**Results:**

High immunoreactivity of phospho-Drp1^Ser637^ was significantly correlated with high-grade serous carcinoma (HGSC) (*p* = 0.034), residual postoperative tumor of > 1 cm (*p* = 0.006), and non-responders to adjuvant chemotherapy (*p* = 0.007), whereas high expression of CaMKI was significantly correlated with stage III/IV [International Federation of Gynecologists and Obstetricians (FIGO)] (*p* = 0.011) and platinum-resistant recurrence (*p* = 0.030). ROC curve analysis showed that Drp1, phospho-Drp1^Ser637^ and CaMKI could significantly detect tumor progression with 0.710, 0.779, and 0.686 of area under the curve (AUC), respectively. The Kaplan-Meier survival curve showed that patients with high Drp1, phospho-Drp1^Ser637^ and CaMKI levels had significantly poorer progression free survival (PFS) (*p* = 0.003, *p* < 0.001 and *p* = 0.017, respectively). Using multivariate analyses, phospho-Drp1^Ser637^ was significantly associated with PFS [*p* = 0.043, hazard ratio (HR) 3.151, 95% confidence interval (CI) 1.039–9.561].

**Conclusions:**

Drp1 and CaMKI are novel potential candidates for the detection and prognosis of epithelial OVCA and as such further studies should be performed to exploit their therapeutic significance.

## Background

Ovarian cancer (OVCA) is the most lethal gynecological malignancy, and ranks fifth as the cause of cancer death among women. The standard treatment is cytoreductive surgery coupled with the treatment of first-line chemotherapy with paclitaxel and carboplatin [1]. Regardless, more than half of the patients treated experience disease recurrence within 2 years, irrespective of the effectiveness of first-line chemotherapy and is associated with poor prognosis. Reliable prognostic biomarkers are therefore needed to aid in patient differential diagnosis and tailored therapeutic alternatives to improve patient survival.

Plasma tumor markers such as carbohydrate antigen 125 (CA125) is widely used for differential diagnosis of ovarian tumor and prognosis, tumor recurrence and the prediction of treatment response [2, 3]. However, CA125 is unstable and their levels are affected by histological subtypes, FIGO stage or physiological conditions; thus, making their utilization questionable [4]. Circulating plasma gelsolin (pGSN) has recently been shown to be effective in detecting early stage OVCA and predicting residual disease compared with CA125; however, a large patient cohort is needed to substantiate these findings [5]. The combination of pGSN and CA125 provided a 100% sensitivity in detecting early stage OVCA [5] thus, providing an evidence that combining multiple tumor markers on a panel could increase OVCA diagnosis and revolutionize treatment. In terms of the prediction of treatment response or prognosis, various genomic, transcriptomic and proteomic biomarkers have been reported [6]. Moreover, the usefulness of imaging modalities such as ^18^F-fluorodeoxyglucose positron emission tomography (^18^F-FDG/PET) which reflect cellular glycolytic metabolism has also been reported, and is believed to be a more accurate prediction tool of chemotherapeutic response than CA-125 [7]. However, the identification of reliable biomarkers applied for all patient is urgently needed.

Mitochondria are highly dynamic organelles, and their fission and fusion fulfill mitochondrial function, including respiration, calcium buffering, apoptosis, and autophagy. Dynamin-related protein 1 (Drp1) is the master regulator of mitochondrial fission. Drp1 is mainly present in the cytoplasm but is translocated into the mitochondrial outer membrane and binds to its partner fission proteins such as mitochondrial fission factor (MFF) or mitochondrial fission 1 protein (Fis1) during mitochondrial fission [8]. Drp1 controls the balance between fission and fusion by their phosphorylation at two distinct serine moieties. Phosphorylation of Ser616 activates Drp1 and induces mitochondrial fission, whereas Drp1 is inactivated via Ser637 phosphorylation, resulting in mitochondrial fusion [9]. The role of Drp1-dependent mitochondrial fission and fusion in apoptotic progression and chemoresistance has been reported in different cancer studies [10]. Few studies have reported the relationship between Drp1 and chemoresistance in OVCA although most of them were in vitro studies using OVCA cells with no reports on their clinical relevance [11–16].

We have previously reported that OVCA cells expressed phospho-Drp1^Ser637^ and are prone to form highly interconnected networks [17]. A calcium mobilizing agent, Saikosaponin-d, suppresses phospho-Drp1^Ser637^ content and calcium/calmodulin–dependent protein kinase I (CaMKI) phosphorylation - which has also been reported to up-regulate Drp1 - leading to mitochondrial fission and subsequently apoptosis [17]. Extending from these in vitro findings, we have examined in this current study the clinical relevance and prognostic impact of Drp1, CaMKI and their phosphorylated forms in epithelial OVCA. Our results could assist in the development of targeted treatment options related to mitochondrial dynamics and calcium signaling in epithelial OVCA patients.

## Methods

### Patients and treatment

This study included 49 patients with primary epithelial OVCA treated between 2012 and 2017 at the Department of Obstetrics and Gynecology, University of Fukui. Formalin-fixed, paraffin-embedded tissue samples for all patients were obtained and analyzed retrospectively. Clinical and pathological factors were evaluated by reviewing medical charts and pathology records. The patients with histologically confirmed epithelial OVCA were included and the definitive histopathological diagnosis was performed by 2 certified pathologists based on the World Health Organization (WHO) classification. The patients’ treatment included a combination of debulking surgery and adjuvant chemotherapy according to the clinical guidelines of the Japan Society of Gynecologic Oncology. Patients were followed-up for at least 24 months after the date of their first visit or until death. The study protocol has been approved by the institutional review board of the University of Fukui Hospital (IRB Number:20180150).

### Tissue samples and immunohistochemistry

**Fig. 1:**
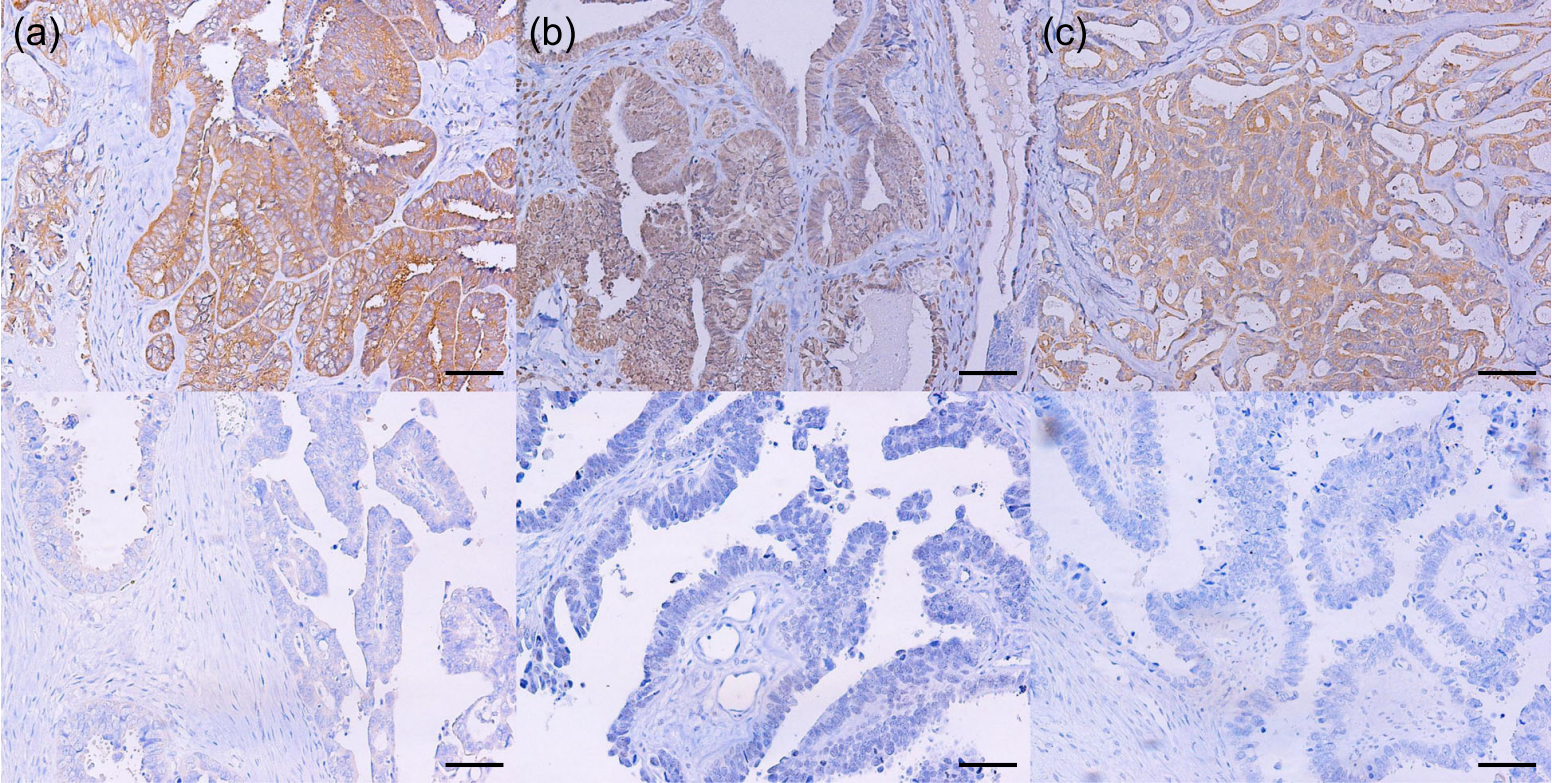
Representative high-grade serous ovarian cancer showing immunostaining for Drp1 (**a**), phospho-Drp1^Ser637^ (**b**), and CaMKI (**c**) (upper) and each negative or weak staining (bottom) (magnification, × 200). Scale bar is 50 μm

Formalin-fixed, paraffin-embedded, 2.5 μm sections were obtained from the samples collected. IHC staining was performed with the avidin–biotin–peroxidase complex method as previously described [18]. Antibodies used in IHC staining are shown in supplementary Table 1. To determine the stain intensity, the stroma was used as internal negative control and the vascular endothelium cells used as internal positive control (supplementary figure 1) [19–21]. The intensity and distribution of the Drp1, phospho-Drp1^Ser637^, CaMKI, and phospho-CaMKI^Thr177^ immunohistochemical staining reaction was evaluated using a semi-quantitative method (IRS-score) as described previously [18]. IRS-score was calculated as follows: IRS = SI x PP, where SI is the optical stain intensity graded as 0 = no, 1 = weak, 2 = moderate, and 3 = strong staining, and PP is the degree of positively stained cells defined as 0 = no staining, 1 = < 10%, 2 = 11–50%, 3 = 51–80%, and 4 = > 81%). Immunohistochemistry staining was scored by 2 independent observers. Representative images of immunostaining are shown in Fig. 1.

### Statistical analysis

The outcome measures were progression-free survival (PFS) and overall survival (OS). PFS was assessed from the date of debulking surgery, whereas OS was from the date of first visit. Tumor progression was confirmed by either tissue biopsy or serial imaging showing evidence of progressive disease. The sample size calculation was performed by the statistical software EZR (Saitama Medical Center, Jichi Medical University, Saitama, Japan) [22] based on the results of CA125 as the predictive marker for PFS [23]. The Mann-Whitney U test was used to analyze relationships between clinical characteristics and IRS-score of each protein. Receiver operating characteristic (ROC) curve analysis was performed to determine optimal cut-off values for discrimination with high accuracy based on the area under the curve (AUC) s for each protein. The Kaplan-Meier curve was used to assess the relationship between tissue markers and PFS and OS; log-rank test was used to calculate the statistical significance. The correlative studies using Pearson were used to determine the correlation among proteins or clinical characteristics. Cox proportional hazards regression modeling was used for univariate and multivariate analyses. Significance was defined as *p* < 0.05 (2-sided testing). All statistical analyses were performed using SPSS Statistics version 24 (IBM, Armonk, NY).

## Results

### Patient characteristics

Clinical information of the 49 patients with their FIGO stages (I, 19; II, 3; III, 20; IV, 7) is summarized in Table 1. The median age at diagnosis was 57.6 years (range, 31–82 years). Twenty patients were < 55 years old and 29 patients were ≥ 55 years old. Histopathological subtypes included high-grade serous carcinoma (HGSC) (*n* = 21), clear cell carcinoma (*n* = 13), endometrioid carcinoma (*n* = 6), mucinous carcinoma (*n* = 6), low-grade serous carcinoma (LGSC) (*n* = 2) and malignant Brenner tumor (*n* = 1). Twenty-nine patients received complete surgery (no postoperative residual tumor), 10 patients received optimal surgery (postoperative residual tumor of less than 1 cm), and 10 patients received suboptimal surgery (postoperative residual tumor of greater than to equal to 1 cm). Forty-three patients (87.8%) received adjuvant chemotherapy with paclitaxel and carboplatin. If the patients had severe neurotoxicity by paclitaxel, docetaxel was used instead of paclitaxel. Six patients (12.2%) did not received adjuvant chemotherapy because of the early stage of disease or the patients’ demand. Based on the findings of computed tomography after last cycle of adjuvant chemotherapy, patients were evaluated for treatment response, which was classified into four categories: complete response when there was resolution of all evidence of disease for at least 1 month; partial response when there was a decrease of ≥50% in the product of the diameters (maximum and minimum) of all measurable lesions without the development of new lesions for at least 1 month; stable disease if there was a decrease of < 50% or an increase of < 25% in the product of the diameters of all measurable lesion; and progressive disease if there was an increase of ≥25% in the product of the diameters of all measurable lesions or the development of new lesions. In the present study, 32 (74.4%) patients who had complete response were considered as responders, whereas 11 (25.6%) patients in the other three categories (partial response, stable disease, and progressive disease) were considered as non-responders [24, 25]. The median follow-up period was 43.5 months (range, 11.7–80.7 months). Twenty-two patients (44.9%) had tumor progression including platinum-sensitive (greater than to equal to 6 months) (16.3%) and –resistant (less than 6 months) (28.6%) during the follow-up period, and 10 patients (20.4%) died.

**Table 1:** Patient and tumor characteristics

Characteristics	n	%
Total number of patients	49	
Age		
< 55	20	40.8
≥ 55	29	59.2
FIGO stage		
I	19	38.8
II	3	6.1
III	20	40.8
IV	7	14.3
Histology		
High-grade serous carcinoma	21	42.9
Non- high-grade serous carcinoma		
Clear	13	26.5
Endometrioid	6	12.2
Mucinous	6	12.2
Low-grade serous carcinoma	2	4.1
Brenner	1	2.0
Completeness of surgical reduction		
Complete	29	59.2
Optimal	10	20.4
Suboptimal	10	20.4
Treatment response to adjuvant chemotherapy		
Responder	32	74.4
Non-responder	11	25.6
Tumor progression	22	44.9
Platinum-sensitive	8	16.3
Platinum-resistant	14	28.6
Death	10	20.4

### Correlation between Drp1, phospho-Drp1^Ser637^, CaMKI, and phospho-CaMKI^Thr177^ expression and clinicopathological parameters

To determine the clinical and prognostic impact of Drp1, CaMKI and their activated forms, their IRS-scores were calculated after IHC staining and correlated with clinicopathological parameters. Their expression was mainly observed in the cancerous lesions compared with the healthy ovarian tissues or the other adjacent organs (supplementary figure 2). The mean IRS-score of Drp1, phospho-Drp1^Ser637^, CaMKI, and phospho-CaMKI ^Thr177^ were 10.71 ± 0.30 (range, 6–12), 5.76 ± 0.44 (range, 1–12), 9.76 ± 0.35 (range, 6–12), and 0.96 ± 0.16 (range, 0–6), respectively. No significant correlations were seen between Drp1 or phospho-CaMKI^Thr177^ and clinical parameters (Table 2). Significant correlations were identified between high expression of phospho-Drp1^Ser637^ and HGSC (*p* = 0.034), suboptimal surgery (*p* = 0.006), and non-responders (*p* = 0.007) (Table 2). Significant correlations were also identified between high expression of CaMKI and both FIGO stage III-IV (*p* = 0.011) and platinum-resistant recurrence (*p* = 0.030). Furthermore, CA125 showed significant correlations with FIGO stage (*p* < 0.001) and HGSC (*p* = 0.003) (Table 2).

**Table 2:** Immunoreactive Score of Drp1, phospho-Drp1^Ser637^, CaMKI, phospho-CaMKI^Thr177^ of the tumor and CA125 in relation to clinical factors of patients with ovarian cancer

Variable	Number of patients	Drp1		phospho-Drp1^Ser637^		CaMKI		phospho-CaMKI^Thr177^		CA125	
		mean ± SE	*p*	mean ± SE	*P*	mean ± SE	*p*	mean ± SE	*p*	mean ± SE	*p*
Age											
< 55	20	10.05 ± 0.52	0.054	4.75 ± 0.66	0.053	9.25 ± 0.55	0.231	1.10 ± 0.24	0.311	792.3 ± 376.3	0.127
≥55	29	11.17 ± 0.35		6.45 ± 0.57		10.10 ± 0.44		0.86 ± 0.21		2141.0 ± 921.6	
FIGO stage											
I-II	22	10.14 ± 0.46	0.050	5.00 ± 0.58	0.133	8.77 ± 0.52	0.011*	0.73 ± 0.19	0.122	340.8 ± 195.6	< 0.001*
III-IV	27	11.19 ± 0.39		6.37 ± 0.63		10.56 ± 0.42		1.15 ± 0.24		2608.7 ± 987.8	
Histology											
High-grade serous carcinoma	21	11.24 ± 0.43	0.098	6.62 ± 0.61	0.034*	9.71 ± 0.52	0.974	1.05 ± 0.26	0.502	2832.5 ± 1243.6	0.003*
Non- high-grade serous carcinoma	28	10.32 ± 0.42		5.11 ± 0.61		9.79 ± 0.48		0.89 ± 0.20		658.9 ± 278.7	
Completeness of surgical reduction											
Complete or optimal	39	10.39 ± 0.36	0.085	5.15 ± 0.46	0.006*	9.46 ± 0.40	0.116	1.03 ± 0.19	0.669	1753.4 ± 712.7	0.264
Suboptimal	10	12.00 ± 0.00		8.10 ± 0.94		10.90 ± 0.57		0.70 ± 0.15		955.0 ± 281.5	
Treatment response											
Responders	32	10.34 ± 0.40	0.209	5.00 ± 0.47	0.007*	9.25 ± 0.44	0.252	1.00 ± 0.23	0.732	1118 ± 381.5	0.117
Non-responders	11	11.45 ± 0.55		7.82 ± 0.86		10.36 ± 0.70		0.82 ± 0.12		3183 ± 2231	
Tumor progression											
No progression or platinum-sensitive	35	10.37 ± 0.38	0.056	5.40 ± 0.52	0.160	9.29 ± 0.43	0.030*	0.89 ± 0.16	0.633	1007.7 ± 300.8	0.101
Platinum-resistant	14	11.57 ± 0.43		6.64 ± 0.80		10.93 ± 0.47		1.14 ± 0.39		3047.4 ± 1839	

* *p* < 0.05

### Clinical performances of Drp1, phospho-Drp1^Ser637^, CaMKI, and phospho-CaMKI^Thr177^ expressions

**Fig. 2:**
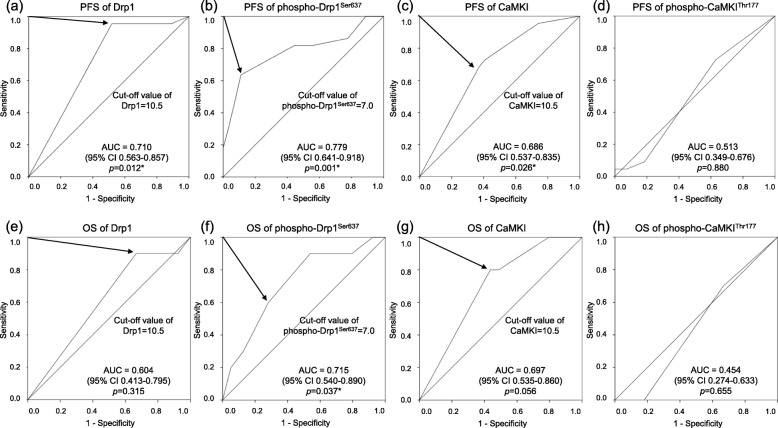
Receiver operating characteristic curve analyses for predicting progression-free survival (**a-d**) and overall survival (**e-h**) according to IRS-score of Drp1, phospho-Drp1^Ser637^, CaMKI and phospho-CaMKI^Thr177^. **a** Area under the curve (AUC) is 0.710 (*p* = 0.012, 95% confidence interval (95%CI) 0.563–0.857), with 10.5 determined as the optimal cut-off for Drp1. **b** AUC is 0.779 (*p* = 0.001, 95%CI 0.641–0.918), and 7.0 is the optimal cut-off for phospho-Drp1^Ser637^. **c** AUC is 0.686 (*p* = 0.026, 95%CI 0.537–0.835), and 10.5 is the optimal cut-off for CaMKI. **d** AUC is 0.513 (*p* = 0.880, 95%CI 0.349–0.676 for phospho-CaMKI^Thr177^. **e** AUC is 0.604 (*p* = 0.315, 95%CI 0.413–0.795), with 10.5 determined as the optimal cut-off for Drp1. **f** AUC is 0.715 (*p* = 0.037, 95%CI 0.540–0.890), and 7.0 is the optimal cut-off for phospho-Drp1^Ser637^. **g** AUC is 0.697 (*p* = 0.056, 95%CI 0.535–0.860), and 10.5 is the optimal cut-off for CaMKI. **h** AUC is 0.454 (*p* = 0.655, 95%CI 0.274–0.633) for phospho-CaMKI^Thr177^

Using Fisher’s test to determine optimal IRS-score cut-offs, ROC curve analysis was used to test and compare the performances of tissues markers under investigation. With a cut-off of 10.5, Drp1 could significantly detect tumor progression, but not overall survival (OS) (area under the curve (AUC), 0.710, 95.5% sensitivity, 48.1% specificity for tumor progression; AUC, 0.604, 90.0% sensitivity, 33.3% specificity for OS). A phospho-Drp1^Ser637^ cut-off of 7.0 was observed to significantly detect both tumor progression and OS (AUC, 0.779, 63.6% sensitivity, 88.9% specificity for tumor progression; AUC, 0.715, 60.0% sensitivity, 71.8% specificity for OS). With a cut-off of 10.5, CaMKI significantly detected tumor progression and demonstrated a tendency for OS (AUC, 0.686, 68.2% sensitivity, 63.0% specificity for tumor progression; AUC, 0.697, 80.0% sensitivity, 56.4% specificity for OS). At a cut-off of 0.5, phospho-CaMK^Thr177^ demonstrated no significant detection for tumor progression and OS (AUC, 0.513, 72.7% sensitivity, 37.0% specificity for tumor progression; AUC, 0.454, 70.0% sensitivity, 33.3% specificity for OS), (Fig. 2).

### Prognostic effect of Drp1, phospho- Drp1^Ser637^, and CaMKI expression

**Fig. 3:**
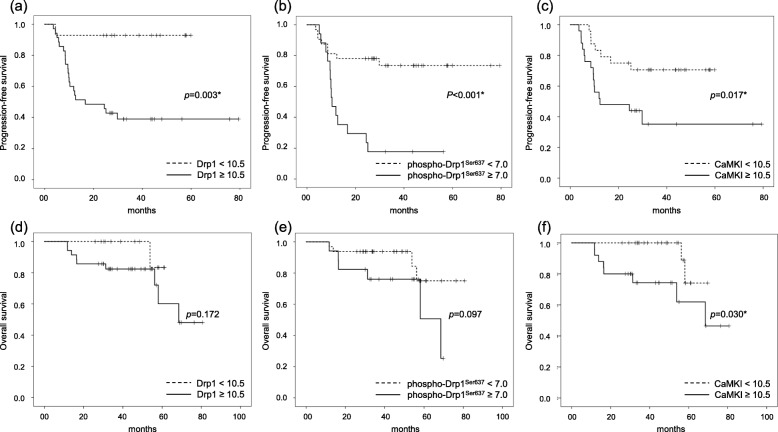
Kaplan-Meier survival curves for progression-free survival and overall survival rates among patients with epithelial ovarian cancer according to IRS-score of Drp1, phospho-Drp1^Ser637^, and CaMKI. **a** PFS in patients with high IRS-score of Drp1 (≥10.5, solid line) and low IRS-score of Drp1 (< 10.5, dotted line). Patients with high IRS-score of Drp1 showed poorer PFS (*p* = 0.003) compared with patients with low IRS-score of Drp1. **b** PFS in patients with high IRS-score of phospho-Drp1^Ser637^ (≥7.0, solid line) and low IRS-score of phospho-Drp1^Ser637^ (< 7.0, dotted line). Patients with high IRS-score of phospho-Drp1^Ser637^ showed poorer PFS (*p* < 0.001) than patients with low IRS-score of phospho-Drp1^Ser637^. **c** PFS in patients with high IRS-score of CaMKI (≥10.5, solid line) and low IRS-score of CaMKI (< 10.5, dotted line). Patients with high IRS-score of CaMKI showed poorer PFS (*p* = 0.017) than patients with low IRS-score of CaMKI. **d** OS in patients with high IRS-score of Drp1 (≥10.5, solid line) and low IRS-score of Drp1 (< 10.5, dotted line). No differences in OS is apparent according to IRS-score of Drp1. **e** OS in patients with high IRS-score of phospho-Drp1^Ser637^ (≥7.0, solid line) and low IRS-score of phospho-Drp1^Ser637^ (< 7.0, dotted line). No differences in OS is apparent according to IRS-score of phospho-Drp1^Ser637^. **f** OS in patients with high IRS-score of CaMKI (≥10.5, solid line) and low IRS-score of CaMKI (< 10.5, dotted line). Patients with high IRS-score of CaMKI show poorer OS (*p* = 0.030) than patients with low IRS-score of CaMKI

We excluded phospho-CaMK^Thr177^ in the subsequent analysis because ROC curve analysis failed to show any significant effect on the tumor progression and survival. Kaplan-Meier survival curves showed that the patients with high expression of Drp1, phospho-Drp1^Ser637^, and CaMKI showed significantly poor PFS (*p* = 0.003, *p* < 0.001 and *p* = 0.017, respectively) compared with patients with low expression. Moreover, patients with high expression of CaMKI showed significantly poorer OS (*p* = 0.030) than those with low expressions (Fig. 3).

Univariate analysis showed that high IRS-score of Drp1, phospho-Drp1^Ser637^, and CaMKI were significantly associated with poor PFS (*p* = 0.018, 0.001, and 0.022, respectively). ≥55 years old, FIGO stage III-IV, HGSC, suboptimal surgery, non-responders, platinum-resistant recurrence, and high values of CA125 were also significantly associated with poor PFS (*p* = 0.041, *p* < 0.001, *p* = 0.013, *p* < 0.001, *p* < 0.001, *p* < 0.001, and *p* = 0.021, respectively; Table 3). In multivariate analysis, since Drp1 and phospho-Drp1^Ser637^ were related variables using Pearson correlative studies (*p* = 0.001), two different models including Drp1 and phospho-Drp1^Ser637^ separately were used. FIGO stage, the completeness of surgical reduction, treatment response to adjuvant chemotherapy, and tumor progression were also related variables (*p* = 0.001). Therefore, FIGO stage was used for multivariate analysis. FIGO stage III-IV (*p* = 0.009) and phospho-Drp1^Ser637^ (*p* = 0.043) were significantly associated with poor PFS and independent prognostic factors for PFS (Table 3).

**Table 3:** Prognostic factors for progression-free survival with ovarian cancer selected by Cox’s uni- and multivariate analysis

Variables	Univariate analysis		Multivariate analysis			
	Hazard ratio (95%CI)	*P*	Hazard ratio (95%CI)	*p*	Hazard ratio (95%CI)	*p*
Age (≥55)	2.830 (1.043–7.682)	0.041*	1.045 (0.234–4.666)	0.954	0.781 (0.163–3.731)	0.757
FIGO stage (III-IV)	13.605 (3.157–58.631)	< 0.001*	7.299 (1.492–35.721)	0.014*	9.094 (1.723–48.010)	0.009*
Histopathologic type (High-grade serous carcinoma)	3.032 (1.268–7.249)	0.013*	1.885 (0.505–7.031)	0.345	1.937 (0.523–7.171)	0.322
Completeness of surgical reduction (Suboptimal)	8.007 (3.239–19.795)	< 0.001*				
Treatment response (non-responders)	16.067 (5.362–48.143)	< 0.001*				
Tumor progression (platinum-resistant)	38.183 (10.233–142.469)	< 0.001*				
CA125 (≥399.3)	2.730 (1.161–6.421)	0.021*	1.176 (0.413–3.350)	0.762	0.686 (0.222–2.114)	0.511
Drp1 (≥10.5)	11.338 (1.521–84.495)	0.018*	4.568 (0.551–37.879)	0.159		
phospho-Drp1^Ser637^ (≥7.0)	4.632 (1.912–11.220)	0.001*			3.151 (1.039–9.561)	0.043*
CaMKI (≥10.5)	2.864 (1.161–7.065)	0.022*	1.809 (0.690–4.744)	0.228	2.740 (0.997–7.532)	0.051

* *p* < 0.05

Univariate analysis showed that suboptimal surgery, non-responders, platinum-resistant recurrence, and CaMKI were significantly associated with poor OS (*p* = 0.015, 0.003, 0.002, and 0.048, respectively). In multivariate analysis, no independent prognostic factor for OS was identified other than suboptimal surgery (*p* = 0.047) (Table 4).

**Table 4:** Prognostic factors for overall survival with ovarian cancer selected by Cox’s uni- and multivariate analysis

Variables	Univariate analysis		Multivariate analysis	
	Hazard ratio (95%CI)	*p*	Hazard ratio (95%CI)	*p*
Age (≥55)	1.094 (0.270–4.441)	0.900		
FIGO stage (III-IV)	7.256 (0.913–57.650)	0.061		
Histopathologic type (High-grade serous carcinoma)	1.487 (0.423–5.223)	0.536		
Completeness of surgical reduction (Suboptimal)	4.691 (1.346–16.343)	0.015*	3.610 (1.020–12.779)	0.047*
Treatment response (non-responders)	11.701 (2.291–59.775)	0.003*		
Tumor progression (platinum-resistant)	12.210 (2.583–57.716)	0.002*		
CA125 (≥461.35)	2.272 (0.651–7.925)	0.198		
Drp1 (≥10.5)	3.847 (0.479–30.881)	0.205		
phospho-Drp1^Ser637^ (≥7.0)	2.797 (0.787–9.942)	0.112		
CaMKI (≥10.5)	4.877 (1.011–23.519)	0.048*	3.844 (0.772–19.145)	0.100

* *p* < 0.05

## Discussion

In the present study, we have demonstrated that the expressions of Drp1 and CaMKI had the most significant prognostic correlations in the patients with epithelial OVCA. Combined with our previous in vitro studies, both Drp1 and CaMKI could serve as potential target proteins for therapeutic purposes as well as possible prognostic biomarkers.

Mitochondria fusion and fission are essential to maintaining healthy mitochondrial function cells and important for many physiological functions, including energy generation, metabolism, calcium signaling and cell death. Drp1 is a member of the dynamin family of guanosine triphosphatases (GTPases) and plays a critical role in the mitochondrial dynamics. Drp1 has also been reported to be associated with the development of cancers by regulating various cellular processes such as cell death, metabolic reprogramming or cell cycle. Although many in vitro studies with various cancer cells on the possible involvement of Drp1 in cancer development and progression have been reported, very few involve an assessment of tissue samples in the context of the clinical outcome on the cancer patients [9, 10]. Rehman et al. have demonstrated that Drp1 was highly expressed in adenocarcinoma lesions compared with healthy lungs in tissue samples from patients with lung cancer [26]. Zhao et al. also observed in breast cancer patients that the expression of Drp1 is proportional to the degree of invasiveness and metastasis [27]. In the present studies, the expression of Drp1 is mainly observed in the cancerous lesions compared with the healthy ovarian tissues; this also correlated with tumor progression suggesting that Drp1 plays a pivotal role in the progression of various cancers including ovarian cancer.

Drp1 controls the balance between fission and fusion by phosphorylation at two distinct serine moieties. Phosphorylation of Ser616 activates Drp1 and induces mitochondrial fission whereas Drp1 is inactivated via Ser637 phosphorylation, resulting in mitochondrial fusion [9]. In some cancers such as lung cancer, breast cancer or melanoma, increased levels of phospho-Drp1^Ser616^ and mitochondrial fission are associated with cancer progression [26–28]. Han Y et al. also reported in ovarian cancer cells that hypoxia promoted mitochondrial fission and cisplatin resistance through down-regulation of phospho-Drp1^Ser616^ [11]. In contrast, we observed that chemoresistant uterine cervical and ovarian cancer cells exhibit highly interconnected mitochondrial networks and that mitochondrial fusion may contribute to chemoresistance [16, 17, 29]. Moreover, we have previously shown that the calcium mobilizing agent Saikosaponin-d suppresses phospho-Drp1^Ser637^ content and CaMKI phosphorylation, leading to mitochondrial fission and subsequent apoptosis [17]. These results are consistent with the study by Yu Y et al. which reported that the inhibitor of anti-apoptotic BCL2 family protein increased the levels of Drp1, mitochondrial fission and apoptosis in cisplatin resistant ovarian cancer cells [13]. Thus, Drp1-dependent mitochondrial dynamics may confer chemosensitivity or resistance depending on the cancer type and in a cell line-specific manner. Therefore, we explored the expression of Drp1, CaMKI and their phosphorylated-form; these were related with clinical and prognostic effects using immunohistochemical analysis of tissue samples collected from patients with epithelial OVCA.

We have explored the correlation of Drp1, phospho-Drp1^Ser637^, CaMKI and phospho-CaMKI^Thr177^ expressions as well as serum CA125 levels with clinicopathological parameters. Age was not correlated with any parameter although most ovarian cancers develop after menopause. High levels of CA125 was significantly associated with advanced FIGO stage and HGSC and these results are consistent with previous report [4]. High expression of phospho-Drp1^Ser637^ but not Drp1 was associated with HGSC, suboptimal surgery and non-responders for adjuvant chemotherapy. High expression of CaMKI was significantly associated with advanced FIGO stage and platinum-resistant recurrence whereas the expression of phospho-CaMKI^Thr177^ was not observed in the present study. In our previous in vitro study, CaMKI was phosphorylated by calcium mobilizing agent, leading to mitochondrial fission. Thus, phospho-CaMKI^Thr177^ was not expressed because the patients were newly diagnosed without any exposure to treatment. Interestingly, phospho-Drp1^Ser637^ and CaMKI were more associated with clinicopathological parameters than Drp1, suggesting that these patients could be stratified and selected for targeted therapy such as mobilizing agents as previously described [17]. However, our results are not consistent with the interactive open-access database which was published in 2017 [19] where CaMKI was not expressed in OVCA tissues. The reason (s) for this apparent difference is not known, it is possible that this could partly be due to the differences of antibodies used for the immunohistological analysis as we used monoclonal antibody for CaMKI detection against their polyclonal antibody. Thus, further studies are needed to investigate the expression patterns of these tissue markers using more reliable antibodies. In terms of patients’ outcome, the expression of Drp1, phospho-Drp1^Ser637^ and CaMKI as well as CA125 were significantly associated with PFS. In particular, phospho-Drp1^Ser637^ emerged as an independent prognostic factor for PFS but not OS. PFS provides insight into the time frame for tumor recurrence and thus plays a key role in chemoresistance. These results are conceivable since high expression of phospho-Drp1^Ser637^ was also associated with suboptimal surgery and non-responders for adjuvant chemotherapy. These findings are consistent with that of Meshach A-W et al, where pGSN mRNA expression was associated with PFS but not OS in ovarian cancer patients [30]. Conversely, none of these proteins presented as independent prognostic factors for OS, a phenomena consistent with other reports [19]. Thus, further investigations are needed to explore novel biomarkers predictive of patient survival.

Although the findings from this study are promising, we also acknowledge some associated limitations. Our study is retrospective and monocentric. Therefore, further prospective and multicenter studies are needed to determine the prognostic value of Drp1, CaMKI and their phosphorylated-forms. We also look forward to validating these findings in larger patient cohorts with diverse histological subtypes.

## Conclusion

We have for the first time provided new insight into the clinical prognostic impact of Drp1 and CaMKI in epithelial OVCA patients. Phospho-Drp1^Ser637^ emerged as an independent prognostic factor for PFS which serves as the first report to explain the role of Drp1 and its related proteins in comprehensive patients’ cohort using immunohistochemistry. These findings are promising and provide important insights into developing novel prognostic marker and targeted therapy in the patients with epithelial OVCA.

## Supplementary information

**Additional file 1: Supplementary figure 1.** Representative high-grade serous ovarian cancer showing immunostaining for the vascular endothelium cells as internal positive control (arrows) and the stroma as internal negative control (magnification, × 200). Scale bar is 50 μm. **Supplementary figure 2.** Representative ovary, corpus uteri, uterine cervix, and omentum showing immunostaining for Drp1, phospho-Drp1^Ser637^, and CaMKI (magnification, × 200). In the ovary, all of them were expressed in the granulosa and theca cells whereas none of them were expressed in the epithelial cells as well as weakly expressed in the primordial follicle. In corpus uteri, Drp1 and CaMKI were strongly expressed in the endometrial glands, whereas phospho-Drp1^Ser637^ were moderately expressed. In uterine cervix, Drp1 and CaMKI were strongly expressed in the cervical glands and squamous epithelium cells, whereas phospho-Drp1^Ser637^ were moderately expressed. In omentum, none of them were expressed except for the vascular endothelium cells. Scale bar is 50 μm.

**Additional file 2: Supplementary Table 1.** Primary antibodies and dilutions used for immunohistochemical analysis.

## Data Availability

All data generated or analyzed during this study are included in this published article and its supplementary information files.

## Abbreviations

Drp1: Dynamin related protein 1
CaMKI: Calcium/calmodulin–dependent protein kinase I
OVCA: Ovarian cancer
IHC: Immunohistochemistry
IRS: Immunoreactive score
HGSC: High-grade serous carcinoma
FIGO: International Federation of Gynecologists and Obstetricians
ROC: Receiver operating characteristic
AUC: Area under the curve
PFS: Progression free survival
HR: Hazard ratio
CI: Confidence interval
CA125: Carbohydrate antigen 125
pGSN: Plasma gelsolin
^18^F-FDG/PET: ^18^F-fluorodeoxyglucose positron emission tomography
MFF: Mitochondrial fission factor
Fis1: Mitochondrial fission 1 protein
WHO: World Health Organization
IRB: Institutional review board
OS: Overall survival
LGSC: Low-grade serous carcinoma
GTPases: Guanosine triphosphatases

## References

[1] Mutch DG, Prat J (2014). 2014 FIGO staging for ovarian, fallopian tube and peritoneal cancer. Gynecol Oncol.

[2] Rustin GJ, Nelstrop AE, McClean P, Brady MF, McGuire WP, Hoskins WJ, Mitchell H, Lambert HE (1996). Defining response of ovarian carcinoma to initial chemotherapy according to serum CA 125. J Clin Oncol.

[3] Rustin GJ, van der Burg ME, Griffin CL, Guthrie D, Lamont A, Jayson GC, Kristensen G, Mediola C, Coens C, Qian W (2010). Early versus delayed treatment of relapsed ovarian cancer (MRC OV05/EORTC 55955): a randomised trial. Lancet.

[4] Nakagawa N, Koda H, Nitta N, Nakahara Y, Uno J, Hashimoto T, Nakahori T, Hasegawa M, Kataoka M (2015). Reactivity of CA19-9 and CA125 in histological subtypes of epithelial ovarian tumors and ovarian endometriosis. Acta Med Okayama.

[5] Asare-Werehene M, Communal L, Carmona E, Le T, Provencher D, Mes-Masson AM, Tsang BK (2019). Pre-operative circulating plasma Gelsolin predicts residual disease and detects early stage ovarian Cancer. Sci Rep.

[6] Pokhriyal R, Hariprasad R, Kumar L, Hariprasad G (2019). Chemotherapy Resistance in Advanced Ovarian Cancer Patients. Biomark Cancer.

[7] Tsuyoshi H, Yoshida Y (2017). Diagnostic imaging using positron emission tomography for gynecological malignancy. J Obstet Gynaecol Res.

[8] Archer SL (2013). Mitochondrial dynamics--mitochondrial fission and fusion in human diseases. N Engl J Med.

[9] Kong B, Tsuyoshi H, Orisaka M, Shieh DB, Yoshida Y, Tsang BK (2015). Mitochondrial dynamics regulating chemoresistance in gynecological cancers. Ann N Y Acad Sci.

[10] Lima Ana Rita, Santos Liliana, Correia Marcelo, Soares Paula, Sobrinho-Simões Manuel, Melo Miguel, Máximo Valdemar (2018). Dynamin-Related Protein 1 at the Crossroads of Cancer. Genes.

[11] Han Youngjin, Kim Boyun, Cho Untack, Park In Sil, Kim Se Ik, Dhanasekaran Danny N., Tsang Benjamin K., Song Yong Sang (2019). Mitochondrial fission causes cisplatin resistance under hypoxic conditions via ROS in ovarian cancer cells. Oncogene.

[12] Yang Z, Feng Z, Gu J, Li X, Dong Q, Liu K, Li Y, OuYang L (2017). microRNA-488 inhibits chemoresistance of ovarian cancer cells by targeting Six1 and mitochondrial function. Oncotarget.

[13] Yu Y, Xu L, Qi L, Wang C, Xu N, Liu S, Li S, Tian H, Liu W, Xu Y (2017). ABT737 induces mitochondrial pathway apoptosis and mitophagy by regulating DRP1-dependent mitochondrial fission in human ovarian cancer cells. Biomed Pharmacother.

[14] Tanwar DK, Parker DJ, Gupta P, Spurlock B, Alvarez RD, Basu MK, Mitra K (2016). Crosstalk between the mitochondrial fission protein, Drp1, and the cell cycle is identified across various cancer types and can impact survival of epithelial ovarian cancer patients. Oncotarget.

[15] Qian W, Wang J, Roginskaya V, McDermott LA, Edwards RP, Stolz DB, Llambi F, Green DR, Van Houten B (2014). Novel combination of mitochondrial division inhibitor 1 (mdivi-1) and platinum agents produces synergistic pro-apoptotic effect in drug resistant tumor cells. Oncotarget.

[16] Farrand L, Kim JY, Im-Aram A, Suh JY, Lee HJ, Tsang BK (2013). An improved quantitative approach for the assessment of mitochondrial fragmentation in chemoresistant ovarian cancer cells. PLoS One.

[17] Tsuyoshi H, Wong VKW, Han Y, Orisaka M, Yoshida Y, Tsang BK (2017). Saikosaponin-d, a calcium mobilizing agent, sensitizes chemoresistant ovarian cancer cells to cisplatin-induced apoptosis by facilitating mitochondrial fission and G2/M arrest. Oncotarget.

[18] Yoshida Y, Kurokawa T, Horiuchi Y, Sawamura Y, Shinagawa A, Kotsuji F (2010). Localisation of phosphorylated mTOR expression is critical to tumour progression and outcomes in patients with endometrial cancer. Eur J Cancer.

[19] Uhlen M, Zhang C, Lee S, Sjostedt E, Fagerberg L, Bidkhori G, Benfeitas R, Arif M, Liu Z, Edfors F, et al. A pathology atlas of the human cancer transcriptome. Science. Genes (Basel). 2018;9(2). 10.1126/science.aan2507 28818916

[20] Tanner MJ, Wang J, Ying R, Suboc TB, Malik M, Couillard A, Branum A, Puppala V, Widlansky ME (2017). Dynamin-related protein 1 mediates low glucose-induced endothelial dysfunction in human arterioles. Am J Physiol Heart Circ Physiol.

[21] Marsboom G, Toth PT, Ryan JJ, Hong Z, Wu X, Fang YH, Thenappan T, Piao L, Zhang HJ, Pogoriler J (2012). Dynamin-related protein 1-mediated mitochondrial mitotic fission permits hyperproliferation of vascular smooth muscle cells and offers a novel therapeutic target in pulmonary hypertension. Circ Res.

[22] Kanda Y (2013). Investigation of the freely available easy-to-use software 'EZR' for medical statistics. Bone Marrow Transplant.

[23] van Altena AM, Kolwijck E, Spanjer MJ, Hendriks JC, Massuger LF, de Hullu JA (2010). CA125 nadir concentration is an independent predictor of tumor recurrence in patients with ovarian cancer: a population-based study. Gynecol Oncol.

[24] Lu L, Risch E, Deng Q, Biglia N, Picardo E, Katsaros D, Yu H (2013). An insulin-like growth factor-II intronic variant affects local DNA conformation and ovarian cancer survival. Carcinogenesis.

[25] Lu L, Schwartz P, Scarampi L, Rutherford T, Canuto EM, Yu H, Katsaros D (2011). MicroRNA let-7a: a potential marker for selection of paclitaxel in ovarian cancer management. Gynecol Oncol.

[26] Rehman J, Zhang HJ, Toth PT, Zhang Y, Marsboom G, Hong Z, Salgia R, Husain AN, Wietholt C, Archer SL (2012). Inhibition of mitochondrial fission prevents cell cycle progression in lung cancer. FASEB J.

[27] Zhao J, Zhang J, Yu M, Xie Y, Huang Y, Wolff DW, Abel PW, Tu Y (2013). Mitochondrial dynamics regulates migration and invasion of breast cancer cells. Oncogene.

[28] Wieder SY, Serasinghe MN, Sung JC, Choi DC, Birge MB, Yao JL, Bernstein E, Celebi JT, Chipuk JE (2015). Activation of the mitochondrial fragmentation protein DRP1 correlates with BRAF(V600E) melanoma. J Invest Dermatol.

[29] Kong B, Wang Q, Fung E, Xue K, Tsang BK (2014). p53 is required for cisplatin-induced processing of the mitochondrial fusion protein L-Opa1 that is mediated by the mitochondrial metallopeptidase Oma1 in gynecologic cancers. J Biol Chem.

[30] Asare-Werehene M, Nakka K, Reunov A, Chiu CT, Lee WT, Abedini MR, Wang PW, Shieh DB, Dilworth FJ, Carmona E, et al. The exosome-mediated autocrine and paracrine actions of plasma gelsolin in ovarian cancer chemoresistance. Oncogene. 2019. 10.1038/s41388-019-1087-9 PMC7018662 31700155

