# Supplementary material for: Prognostic impact of Dynamin related protein 1 (Drp1) in epithelial ovarian cancer
Source: BMC Cancer. 2020 May 24;20:467. doi: 10.1186/s12885-020-06965-4 (PMC7247242; doi:10.1186/s12885-020-06965-4)
Supplement: Supplementary file 1 — Additional file 1: Supplementary figure 1. Representative high-grade serous ovarian cancer showing immunostaining for the vascular endothelium cells as internal positive control (arrows) and the stroma as internal negative control (magnification, × 200). Scale bar is 50 μm. Supplementary figure 2. Representative ovary, corpus uteri, uterine cervix, and omentum showing immunostaining for Drp1, phospho-Drp1Ser637, and CaMKI (magnification, × 200). In the ovary, all of them were expressed in the granulosa and theca cells whereas none of them were expressed in the epithelial cells as well as weakly expressed in the primordial follicle. In corpus uteri, Drp1 and CaMKI were strongly expressed in the endometrial glands, whereas phospho-Drp1Ser637 were moderately expressed. In uterine cervix, Drp1 and CaMKI were strongly expressed in the cervical glands and squamous epithelium cells, whereas phospho-Drp1Ser637 were moderately expressed. In omentum, none of them were expressed except for the vascular endothelium cells. Scale bar is 50 μm. [file 12885_2020_6965_MOESM1_ESM.pptx]

## Slide 1
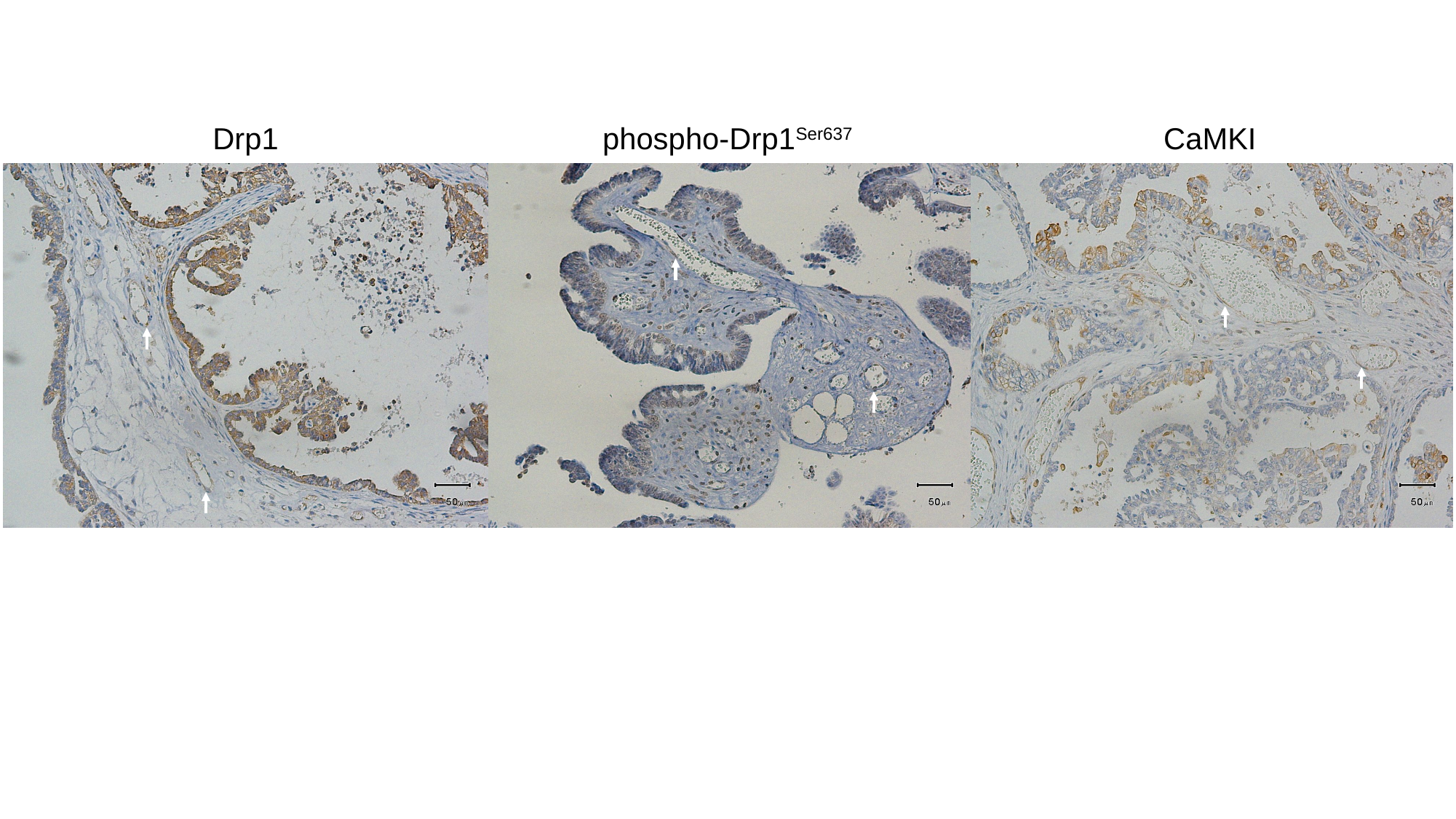

Drp1
phospho-Drp1Ser637
CaMKI

## Slide 2
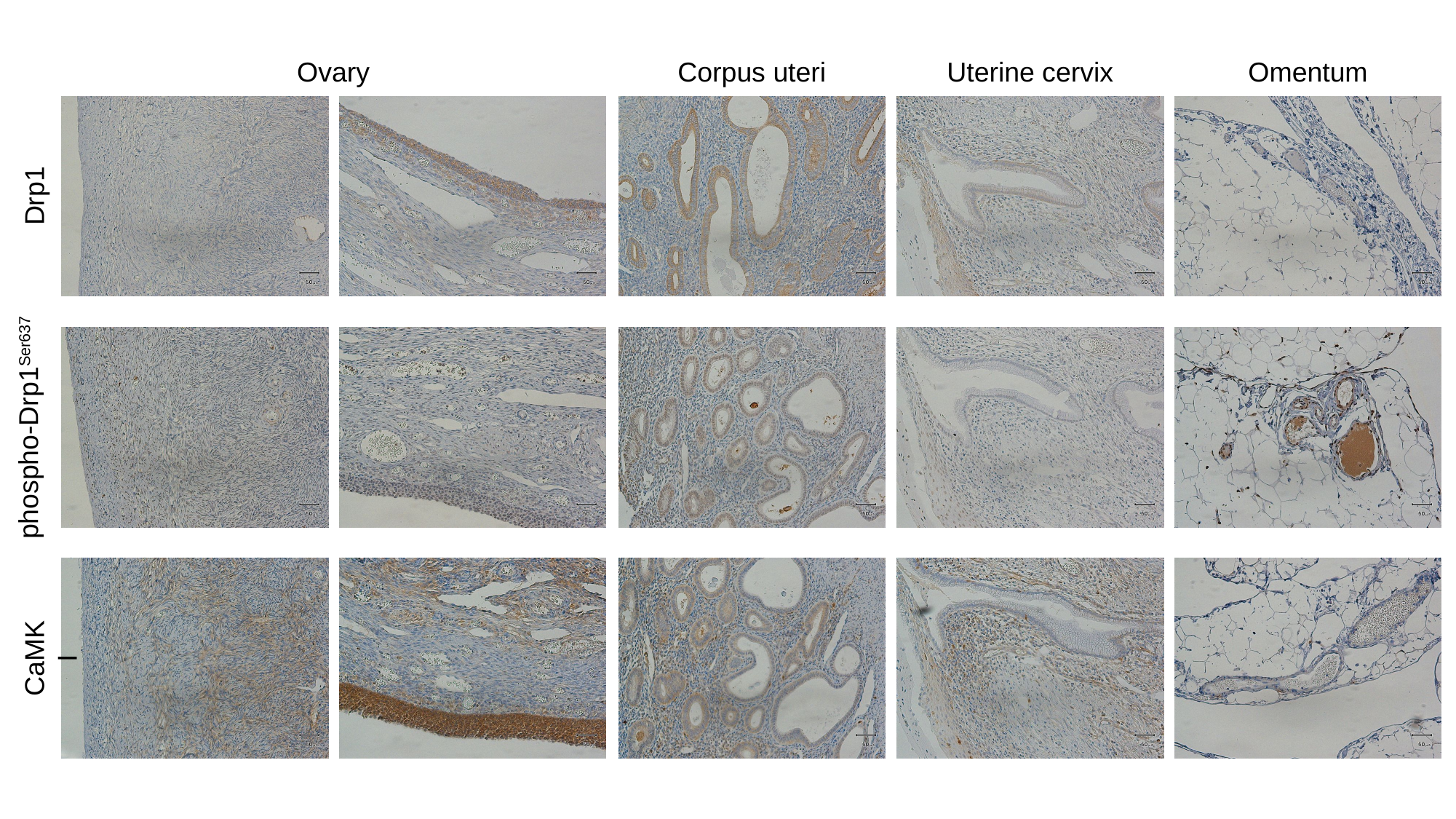

Ovary
Corpus uteri
Uterine cervix
Omentum
Drp1
phospho-Drp1Ser637
CaMKI
