# Supplementary material for: Prognostic impact of Dynamin related protein 1 (Drp1) in epithelial ovarian cancer
Source: BMC Cancer. 2020 May 24;20:467. doi: 10.1186/s12885-020-06965-4 (PMC7247242; doi:10.1186/s12885-020-06965-4)
Supplement: Supplementary file 2 — Additional file 2: Supplementary Table 1. Primary antibodies and dilutions used for immunohistochemical analysis. [file 12885_2020_6965_MOESM2_ESM.docx]

Supplementary Table 1. Primary antibodies and dilutions used for immunohistochemical analysis.

| Antigen | Source | company | Catalogue # | Dilution |
| --- | --- | --- | --- | --- |
| Drp1 | Mouse monoclonal | abcam | ab156951 | 1:50 |
| phospho-Drp1^Ser637^ | Rabbit polyclonal | Biorbyt | orb313185 | 1:400 |
| CaMKI | Rabbit monoclonal | abcam | ab68234 | 1:100 |
| phospho-CaMKI^Thr177^ | Rabbit polyclonal | abcam | ab62215 | 1:250 |
